# Management of Glucose Control in Noncritically Ill, Hospitalized Patients Receiving Parenteral and/or Enteral Nutrition: A Systematic Review

**DOI:** 10.3390/jcm8070935

**Published:** 2019-06-28

**Authors:** Céline Isabelle Laesser, Paul Cumming, Emilie Reber, Zeno Stanga, Taulant Muka, Lia Bally

**Affiliations:** 1 Department of Diabetes, Endocrinology, Clinical Nutrition, and Metabolism, Inselspital, Bern University Hospital, University of Bern, 3010 Bern, Switzerland; 2 Department of Nuclear Medicine, Inselspital, Bern University Hospital, University of Bern, 3010 Bern, Switzerland; 3 School of Psychology and Counselling and IHBI, Queensland University of Technology, Brisbane, QLD 4059, Australia; 4 Institute of Social and Preventive Medicine, University of Bern, 3012 Bern, Switzerland

**Keywords:** glucose control, hyperglycemia, parenteral nutrition, enteral nutrition, nutritional support, insulin

## Abstract

Hyperglycemia is a common occurrence in hospitalized patients receiving parenteral and/or enteral nutrition. Although there are several approaches to manage hyperglycemia, there is no consensus on the best practice. We systematically searched PubMed, Embase, Cochrane Central, and ClinicalTrials.gov to identify records (published or registered between April 1999 and April 2019) investigating strategies to manage glucose control in adults receiving parenteral and/or enteral nutrition whilst hospitalized in noncritical care units. A total of 15 completed studies comprising 1170 patients were identified, of which 11 were clinical trials and four observational studies. Diabetes management strategies entailed adaptations of nutritional regimens in four studies, while the remainder assessed different insulin regimens and administration routes. Diabetes-specific nutritional regimens that reduced glycemic excursions, as well as algorithm-driven insulin delivery approaches that allowed for flexible glucose-responsive insulin dosing, were both effective in improving glycemic control. However, the assessed studies were, in general, of limited quality, and we see a clear need for future rigorous studies to establish standards of care for patients with hyperglycemia receiving nutrition support.

## 1. Introduction

Hyperglycemia is frequently encountered during parenteral (PN) and/or enteral (EN) nutrition in hospitalized patients with and without pre-existing diabetes [1,2]. Indeed, it is estimated that more than 50% of patients on PN and 30% of patients on EN experience hyperglycemia whilst in the hospital [3,4]. Hyperglycemia arises in these patients due to one or more of the following factors: (1) diminished insulin sensitivity due to inflammation, stress hormones, and sedentarism [5]; (2) increased carbohydrate provision [6]; and (3) side-effects of medication such as glucocorticoids that interfere with glucose metabolism [7]. In patients totally reliant on PN, these factors are compounded by the loss of the physiological incretin effect on insulin release, as occurs when entirely bypassing the gastrointestinal tract with intravenous nutrient supply [8]. Furthermore, the diminished glucose-stimulated insulin secretion in diabetic patients with some residual beta-cell function increases their requirement for exogenous insulin.

Evidence from several observational studies suggests that emergent hyperglycemia during nutrition support is associated with increased morbidity and mortality [1,9]. There is an apparently linear relationship between the incidence of adverse outcomes and mean glucose levels once glycaemia surpasses a threshold of 6.3 mM [10]. In individuals on PN, the risk of any complication increases by a factor of 1.58 for each 1 mM increase in glycaemia above this threshold [11]. Conversely, treatment of hyperglycemia is shown to improve clinical outcomes [12,13,14,15]. However, striving for tight glucose control inherently increases the risk of hypoglycemia, which is similarly associated with adverse clinical outcomes [16,17].

PN and EN nutrition support are provided in a number of ways, ranging from continuous to cyclic regimens, often in combination with unpredictable and variable oral intake or additional intravenous glucose administration. Maintaining glycemic control is even more demanding in patients with unanticipated interruptions of their feeding (e.g., due to emergency surgery), or if nutrition support is suspended due to accidental removal or obstruction of tubing. Guidelines such as those from the American Diabetes Society [18] and the Endocrine Society [19] recommend that random blood glucose levels be maintained below 10.0 mM, provided that this target can be safely achieved. According to these guidelines, the mainstay of hyperglycemia management in the hospital is the administration of insulin, given its high efficacy, flexibility, and lack of interference with most other pharmacotherapies or organ dysfunctions.

**Figure 1:**
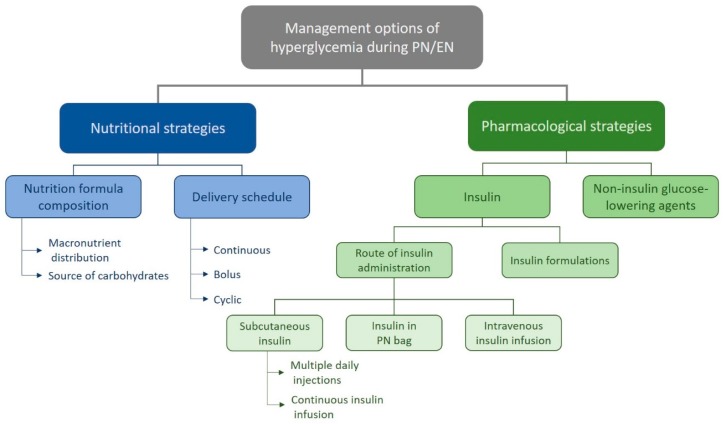
Management options of hyperglycemia during PN/EN. PN = parenteral nutrition, EN = enteral nutrition.

Although there are some recommendations for insulin dosing tailored to the needs of patients receiving PN and/or EN, there is a lack of evidence-based support for specific insulin regimens. Insulin can be delivered via intravenous or subcutaneous routes, or in patients receiving PN, insulin may simply be mixed in the nutrition solution [20]. Intravenous insulin infusion at a rate continuously adjusted according to regular capillary blood glucose measurements helps to maintain glucose levels within the recommended limits. However, implementation of intravenous protocols imposes considerable demand on nursing staff, calling for two hours of direct nursing daily per patient [21], which substantially increases the workload of ward staff. Noncritical care nurses have to manage several patients, and staff levels are reduced at nighttime, which does not encourage constant vigilance of blood glucose and manual adjustment of insulin infusion rates. Thus, for practical and safety considerations in noncritical care settings, subcutaneous administration is the favored route for insulin delivery. However, the formulation of PN and EN support can also influence glucose levels in patients on nutrition support. Whereas glucose is the only carbohydrate source in standard PN solutions, the glycemic impact can be modulated by changing the caloric contributions of carbohydrates versus monounsaturated fatty acids (MUFAs), or by using alternate carbohydrates such as fructose. Furthermore, the addition of fibers to EN formulae can delay carbohydrate absorption, thereby attenuating the glycemic impact [22,23]. An overview of the different management strategies is provided in Figure 1.

Diabetes technology has progressed greatly over the past decade, bringing considerable improvements to the care of outpatients with type 1 and type 2 diabetes. Notably, recent development of continuous glucose monitoring (CGM) systems that measure interstitial fluid glucose concentration every few minutes allow to depict glucose profiles in higher resolution, thereby facilitating adjustment of insulin dosages [24]. CGM systems also project trends for glucose levels and feature customizable hypo- and hyperglycemia alerts. There is a growing interest in the use of this technology in hospital settings given the abundance of additional information that can guide therapy adjustments.

An important variable in the control of glycaemia is the modulation of insulin to meet with the continuously changing metabolic needs. Due to its inherent flexibility of insulin dose adjustment, the predominant mode of delivery in patients with type 1 diabetes is via a subcutaneous insulin pump, also known as continuous subcutaneous insulin infusion [25]. Here, a portable pump infuses rapid-acting insulin at a rate that can be altered on demand or preset to change at fixed times. This flexible adaptation of the insulin delivery profile makes the approach particularly attractive for patients under cyclic nutrition regimens who may receive large amounts of carbohydrates within predefined time windows. The combination of real-time glucose measurements from a CGM device with a control algorithm that directs insulin delivery via an insulin pump constitutes a closed-loop system, also known as the artificial pancreas [26,27]. Closed-loop systems automatically adjust insulin delivery every 10–12 min according to real-time glucose measurements. The autonomy and glucose feedback-regulation obtained through closed-loop systems hold promise in the particular context of hyperglycemia, arising along with nutritional support, while sparing an excess burden on nursing staff.

Our aim in the present review is to provide an overview of the current status and future outlook of glucose management strategies for noncritical care patients receiving PN and/or EN nutrition support.

## 2. Materials and Methods

### 2.1. Literature Search

This review was conducted and reported in accordance with the PRISMA and MOOSE guidelines [28,29]. Completed checklists can be found in Supplementary Tables S5 and S6. PubMed and Embase databases were used to identify relevant records over the past 20 years. The Cochrane Central Register of Controlled Trials and ClinicalTrials.gov were searched for published and ongoing studies. The search strategies for the databases are summarized in Supplementary Tables S1–S4. Two reviewers independently evaluated the titles and abstracts according to the selection criteria. For each potentially eligible study, two reviewers assessed the full-text. In cases of disagreement, a decision was made by consensus or, if necessary, a third reviewer was consulted.

### 2.2. Study Selection Criteria

**Figure 2:**
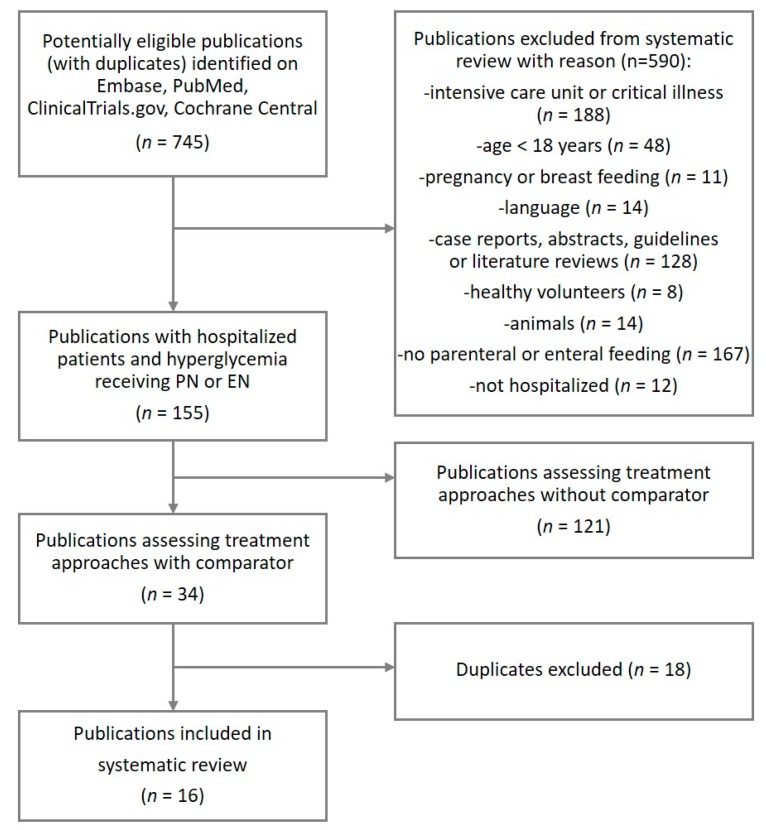
Flowchart illustrating the study selection process.

Studies were included if they met the following criteria: (i) were published or registered in English language between 10 April 1999 and 10 April 2019 (date last searched); (ii) were clinical studies evaluating new treatment approaches against a comparator; (iii) included adult (age ≥ 18 years) noncritical care inpatients receiving PN and/or EN nutrition; (iv) investigated strategies to manage blood glucose control; and (v) had outcomes reflecting glucose control. Exclusion criteria were pregnancy, breast feeding, case reports, abstracts, guidelines, or literature reviews. The selection process is shown in Figure 2.

### 2.3. Data Extraction

Two reviewers extracted data independently using a predesigned form, including study design, sample size, glucose management strategy, primary outcome, and main results. If no primary outcome was specified, we obtained the endpoints deemed most relevant.

### 2.4. Quality Assessment

The quality of clinical trials was evaluated by two reviewers based on the “Cochrane Risk of Bias tool” [30]. According to this tool, studies are judged to be of low or high risk for bias based on criteria to evaluate random sequence generation, allocation concealment, blinding of participants/personnel and outcome assessment, incomplete outcome data, and selective reporting. Since retrospective publications have an inherently high risk of bias in most domains, and there is not a standardized assessment tool available, we refrained them from quality evaluation.

## 3. Results

### 3.1. Study Identification and Selection

We identified in total 745 potentially eligible records. Following screening based on titles and abstracts, 34 citations were selected for detailed full-text evaluation. Of those, 16 articles met the selection criteria and were included in the review (Figure 2). One ongoing study was eligible for inclusion.

### 3.2. Characteristics of the Included Studies

The included published publications comprised 1170 participants from 15 clinical studies, of which 11 were clinical trials (9 randomized and 2 nonrandomized) and 4 were retrospective observational studies. One study was an ongoing registered study. Seven studies were conducted in Europe, three in North America, and five in Asia. Only three studies included more than one center. The study population represented medical (20%), surgical (27%), or mixed medical and surgical (53%) noncritical care inpatients. Three studies explicitly stated the inclusion of patients with type 1 diabetes, three studies explicitly excluded patients with type 1 diabetes, five studies exclusively recruited patients with type 2 diabetes, whereas the remainder did not further characterize the diabetic state of study participants or the reason for hyperglycemia (*n* = 4). Nutrition support comprised PN in six studies, EN in seven studies, and PN and/or EN in two studies. Regarding glucose management strategies, 4 studies investigated nutritional approaches, and 11 studies explored insulin interventions. No studies were found that explored noninsulin pharmacological strategies. The study duration ranged from 36 h to 46 days. Study characteristics and findings are summarized in Table 1 and Table 2.

Four of the clinical trials were considered of medium quality, indicating a low risk of bias in all domains except for performance and detection bias, which was deemed reasonable with a near impossibility of blinding [31,32,33,34]. The other studies were deemed to be of limited quality, with high risk of bias in the domains of lack of primary outcome definition and selective or incomplete reporting (Supplementary Figure S1). Since retrospective publications have an inherently high risk of bias in most domains, we refrained from a quality evaluation of the four such studies.

### 3.3. Studies Examining Nutritional Strategies

Four studies examined nutritional strategies to manage glycaemia in patients receiving nutrition support. The use of EN formulae with lower glycemic impacts were contrasted against conventional products in two clinical trials [35,36]. In a nonrandomized crossover study involving inpatients with type 2 diabetes, Tiyapanjanit et al. tested a formula compounded in-house to contain 50% of calories as carbohydrates, thereof 67% as fructose, in comparison with an iso-energetic control formula (53% of calories as carbohydrates and 15% as fructose). Over the study period of 36 h, lower mean glucose was achieved with the high-fructose formula compared to control (6.8 ± 1.5 vs. 8.0 ± 2.1 mM, *p* = 0.022). No insulin or antidiabetic medication was administered in these patients [35]. Similarly, a four-center randomized controlled parallel trial conducted in 104 inpatients with type 2 diabetes found a lower nutrition-induced relative change from baseline glycaemia with the use of lower glycemic impact enteral vs. standard enteral nutrition formula (10% vs. 21%, *p* = 0.006). The investigated EN formula contained a reduced amount of carbohydrates (9.4 vs. 12.5 g/100 mL, *p* = n/a) and higher amounts of MUFAs (3.8 vs. 1.0 g/100 mL, *p* = n/a) [36]. Similarly, an ongoing randomized clinical trial (GlyENStroke, NCT03422900) evaluates the efficacy of a diabetes-specific enteral nutrition formula to reduce hyperglycemia (glucose levels > 8.3 mM) in nondiabetic patients with hyperglycemia on nutrition support after stroke [37].

There was only one study examining a modified nutritional formula in abdominal surgery patients that were entirely parenterally fed. In this randomized parallel double-blind single-center study, Valero et al. contrasted a PN formula with glucose as a sole carbohydrate source with an iso-energetic PN formulation containing a 2:1:1 glucose:fructose:xylitol carbohydrate mixture. The study population consisted of patients with type 1 diabetes (21%) or type 2 diabetes (79%). Both formulae provided a similar total amount of carbohydrates (2.9 ± 0.5 vs. 2.9 ± 0.7 g/kg/day), but the test formula under investigation contained less glucose (2.9 ± 0.5 vs. 1.5 ± 0.4 g/kg/day). Protein and fat quantity were similar. The time until attaining target glycaemia (8.3–11.1 mM) did not significantly differ (2.5 ± 1.7 vs. 2.4 ± 2.1 day, *p* = ns), nor did the percentage of patients attaining values below 11.1 mM (75% vs. 85%, *p* = ns) and total daily insulin dose (45 ± 19 vs. 45 ± 26 U/day, *p* = ns). However, when stratified according to the occurrence of sepsis, the nonseptic patients showed lower insulin requirements with the glucose–fructose–xylitol regime (37 ± 17 vs. 44 ± 17 U/day, *p* = 0.026) [41].

Managing glycaemia during PN is more demanding than during EN. In this context, the combination of PN with EN, in addition to its well-established trophic benefits on gastrointestinal function, may also confer glycemic advantages. This was shown by a randomized controlled parallel study performed by Lidder et al. in patients undergoing esophagectomy who received nutrition support for up to five days post-surgery. The study contrasted the coverage of 30% of energy needs by EN and 70% by PN feeds with 100% coverage by PN. Although no effect was seen in mean glucose over the five-day study period, the combined use of EN and PN lead to lower glycaemia three days after surgery (*p* = 0.002) [34].

### 3.4. Studies Examining Insulin Strategies

Eleven studies examined insulin-based strategies to manage glucose in patients receiving nutrition support (five with PN, five with EN, and one with combined PN/EN). A randomized controlled single-center parallel study involving 212 type 2 diabetic patients who had undergone gastrectomy for gastric cancer compared protocol-driven intravenous insulin therapy with conventional subcutaneous sliding scale insulin delivery over 8–10 days of continuous EN. Mean glucose levels were lower (5.4 ± 1.2 vs. 9.5 ± 1.8 mM, *p* < 0.001), and mean daily insulin doses were higher (55 ± 15 vs. 32 ± 16 U/day, *p* < 0.001) in patients receiving intravenous insulin compared with subcutaneous insulin. However, in the group receiving intravenous insulin, eight participants experienced episodes of severe hypoglycemia (defined as blood glucose < 2.2 mM), versus only one participant in the subcutaneous group (*p* = 0.010). Additionally, this study reported outcomes extending beyond glycemic control, finding a reduced incidence of surgical site infection in the intravenous insulin group [12].

A second randomized controlled trial (RCT) evaluating different insulin treatments during EN included 50 patients with non-type 1 diabetes who were randomly assigned to receive sliding scale subcutaneous regular insulin either with or without once daily subcutaneous glargine. In the group without glargine administration, subcutaneous neutral protamine Hagedorn (NPH, isophane) was given as a rescue medication when glucose levels exceeded 10.0 mM. Mean glucose as the primary outcome was comparable between groups, as were the number of hypoglycemic events and total daily insulin dose. However, NPH was required in 48% of the control participants, as their glucose levels were not sufficiently controlled with the regular sliding scale subcutaneous insulin alone [40].

A single-arm trial evaluated the efficacy of a computerized variable rate insulin infusion rate protocol in previously insulin-naïve stroke patients on continuous EN over five days. Compared to a historical control population who received enteral bolus feeds accompanied by intravenous insulin coverage, the intervention resulted in a higher percentage of values in the target range of 4.4–6.1 mM (55% vs. 19%, *p* < 0.005) [39].

Additionally, two retrospective observational studies evaluated insulin-based strategies to treat hyperglycemia in hospitalized patients receiving EN. Hijaze et al. found comparable mean glucose values, and similar times with glucose values within the target range (7.8–10 mM), in patients receiving subcutaneous NPH insulin thrice daily vs. basal–bolus insulin therapy with insulin analogues [43]. In the second such study, Hsia et al. retrospectively evaluated glucose control using three different insulin regimens in patients with diabetes of type not otherwise specified during at least three days of continuous EN. Treatment consisted of (1) 70/30 biphasic insulin (NPH/regular) every 8 h (*n* = 6), (2) 70/30 biphasic insulin every 12 h (*n* = 8), and (3) a basal–bolus regimen (glargine and lispro, *n* = 8). The 8 h 70/30 biphasic insulin group had the highest proportion of glucose values falling within the target range (7.8–10.0 mM) (69% vs. 22% vs. 24%, *p* < 0.01). Hypoglycemic events (<3.9 mM) occurred five times in glargine/lispro group, twice in the twice-daily biphasic group, and once in the thrice-daily biphasic group [45]. In patients receiving PN, two randomized controlled parallel trials evaluated strategies that involved the addition of regular insulin to the PN feeding bag. Olveira et al. recently compared the use of 100% coverage of insulin needs by regular insulin added to the PN bag with 50% coverage by regular insulin added to the PN bag and 50% administered subcutaneously as insulin glargine in a total of 161 mixed surgical and medical patients with type 2 diabetes recruited at 26 different sites. The glucose values of participants were evaluated while receiving total PN (maximum of 15 days) and two days after cessation of total PN. Mean glucose levels during total PN did not significantly differ between groups (9.2 ± 2.0 vs. 9.6 ± 2.4 mM, *p* = ns); however, mean glucose two days after cessation of total PN was higher in the group that had received 100% of their exogenous insulin requirements added to the PN feeding bag (8.9 ± 2.5 vs. 7.9 ± 2.4 mM, *p* = 0.024). The authors did not provide any details on the requirement for insulin therapy after cessation of nutrition support [32]. Another randomized controlled clinical trial of PN patients contrasted two different basal insulin regimens. Hakeam et al. compared subcutaneous basal–bolus therapy (insulin glargine + short-acting insulin analogue) with the addition of regular insulin to the PN bag in a total of 67 non-type 1 diabetic patients. Both groups received additional corrective regular subcutaneous insulin according to a sliding scale. Basal insulin delivery dose was titrated based on glucose values by a daily 40%–60% dose increase if glucose values were still above target. The percentage of glucose values within the glycemic target range (7.8–10.0 mM) tended to be higher in the group who received s.c. insulin glargine compared to those who received regular insulin added to the PN bag (52% vs. 48%, *p* = 0.06). Mean glucose levels and number of hypoglycemic events did not differ between treatment groups [33].

In contrast to these prospective findings in PN patients, a retrospective evaluation performed by Truong et al. in 102 patients on PN showed superior glucose control defined as percentage of patients with ≥5 of 6 glucose values <10 mM over 2 days in those who had received 100% of required insulin added to the PN bag (*n* = 78), compared with those treated with subcutaneous insulin glargine (*n* = 35) (72% vs. 49%, *p* = 0.017). Additionally, fewer patients receiving insulin via the PN bag experienced two or more hypoglycemic events compared to those with subcutaneous administration (9% vs. 17.1%, *p* = ns) [42].

As reviewed above in patients receiving EN, a protocol-driven intravenous insulin delivery approach has proven to confer glycemic benefits in PN patients, according to a retrospective analysis performed by Neff et al. A total of 53 surgical and medical patients requiring insulin therapy whilst in the hospital were treated either with protocol-driven variable rate intravenous insulin (*n* = 32), or received basal–bolus subcutaneous insulin therapy (*n* = 21). The insulin infusion group compared to the group receiving subcutaneous basal–bolus insulin therapy showed lower mean glucose levels (9.6 ± 2.1 vs. 11.2 ± 2.6 mM, *p* = 0.009) and a higher percentage of glucose values within the glycemic target of 4.0–10.0 mM (62% vs. 43%, *p* = 0.008), without increased risk of hypoglycemia [44].

Two recently performed randomized controlled parallel design trials evaluated insulin pump therapy, also known as continuous subcutaneous insulin infusion (CSII), in patients receiving nutrition support. The study performed by Li et al. compared the use of CSII (*n* = 50) to basal–bolus therapy using insulin glargine in combination with insulin aspart (*n* = 52) in patients receiving PN. Study treatment was initialized before surgery, and PN began on day 1 after surgery, with comparison of glucose control with CGM from postoperative day 1 to day 5. Glycemic variability was assessed by mean amplitude of glucose excursion (MAGE) as the primary outcome. CSII reduced glycemic variability compared to basal–bolus injection therapy (3.7 ± 2.8 vs. 6.2 ± 3.0 mM, *p* < 0.05). No hypoglycemic events occurred in either treatment group [38].

**Figure 3:**
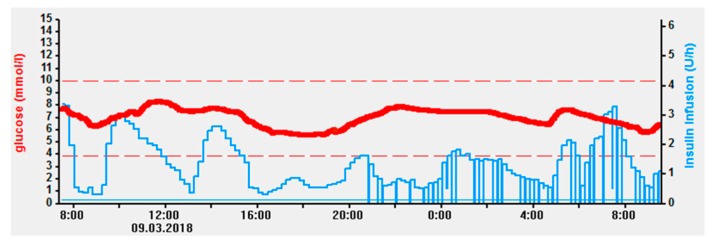
Profile of fully automated subcutaneous closed-loop insulin delivery over 24 h in a noncritical care patient [31]. A control algorithm modulates subcutaneous insulin delivery via an insulin pump (denoted in blue) according to interstitial sensor glucose values (denoted in red). (Kindly provided by Professor Roman Hovorka, University of Cambridge, UK).

The second study evaluated CSII as part of a fully automated subcutaneous closed-loop glucose control (*n* = 21) against conventional subcutaneous insulin therapy according to local practice (*n* = 22) in two different hospitals. Randomization was stratified according to BMI, prestudy total daily insulin dose, and type of nutrition support to ensure to demographic balance between groups. The closed-loop system consisted of a subcutaneous insulin pump, a CGM device, and a control algorithm, which adjusted insulin delivery every 12 min based on real-time CGM values. An example of such fully automated closed-loop insulin delivery is illustrated in Figure 3. Participants were recruited from medical and surgical wards and received PN (*n* = 13), EN (*n* = 27), or combined PN/EN (*n* = 3). The primary outcome was the proportion of time when sensor glucose was within the target range (5.6–10.0 mM). Participants were followed for up to 15 days or until hospital discharge. The closed-loop system nearly doubled the proportion of time spent in the glycemic target range compared to control (68% ± 16% vs. 36% ± 27%, *p* < 0.0001). Time spent above target, mean glucose level, and glucose variability were all significantly lower in the closed-loop group. Hypoglycemia was infrequent in both arms, and its incidence did not differ significantly. The substantially better glycemic control in the closed-loop compared to the control group was achieved with a similar total daily insulin dose (53.9 vs. 40.3 U, *p* = ns) [31].

## 4. Discussion

Hyperglycemia is a common occurrence in hospitalized patients receiving PN and/or EN, and its management in noncritical care settings is challenging. The present review summarized the available evidence for strategies to improve glucose control in this vulnerable population. Improved glucose control can be achieved either by lowering the glycemic impact of nutrition supply and/or matching the nutrition-induced glycemic excursions with a tailored pharmacokinetic profile of a given insulin preparation. Both approaches have been evaluated in a limited number of randomized and nonrandomized clinical trials and observational studies over the past 20 years.

Altering the macronutrient distribution (an increase of calories as MUFAs at the expense of carbohydrates) and the use of nonglucose carbohydrate sources along with high fiber content to delay absorption in EN formulae have proven effective in various studies as well as in meta-analyses [22,23]. The use of diabetes-specific enteral formulae is therefore supported by the expert group of the European Society of Clinical Nutrition and Metabolism (ESPEN) for patients with a history of diabetes [46]. With respect to PN formulae, glucose substitutes such as fructose or xylitol to lower the glycemic impact are no longer used in clinical practice. This may relate to previously reported metabolic side effects of parenteral xylitol and fructose such as formation of oxalate crystals in the kidney and lactate accumulation [47,48,49]. To make matters worse, life-threatening metabolic complications can occur in patients with undeclared hereditary fructose intolerance [50]. There is currently no data on the efficacy and safety of PN formulations with reduced carbohydrate content (and consequently higher protein or lipid fractions) in noncritically ill patients with hyperglycemia. In critically ill patients, however, lipid-based, compared to iso-energetic glucose-based, PN formulae showed more favorable metabolic effects [51]. Additionally, lowering the overall carbohydrate and energy provision has proven effective in reducing hyperglycemia in critically ill patients [52,53].

As an alternative to conventional routes of insulin administration, algorithm-driven intravenous insulin titration protocols during both PN and EN achieved superior glucose control compared to subcutaneous approaches with either sliding scale or basal–bolus insulin therapy. However, this approach poses logistical challenges for ward staff, given the need for frequent glucose draws and insulin dose adjustments, which are either impractical or simply unfeasible in noncritical care settings with low nurse-to-patient ratios. Regarding subcutaneous insulin regimens, administering basal plus supplemental short-acting insulin analogues showed superior efficacy compared to a sliding scale approach with short-acting insulin. The administration of intermediate or long-acting insulins can thus be recommended for PN/EN patients receiving subcutaneous insulin therapy. No data exist with regard to the use of recently introduced ultra-long acting insulins (e.g., degludec). Of note, the required time to reach steady state insulin levels with ultra-long acting formulations imposes certain constraints on the titration method, and prediposes to dysglycemia. There is a practical consideration that administering long-acting insulin in previously insulin-naïve patients brings a risk of hypoglycemia if feeding tubes are accidentally pulled or obstructed.

The admixture of insulin into the PN feeding bag is a safe and effective alternative to using short- or rapid-acting subcutaneous insulin. Moreover, further advantages lie in the lesser need for nursing time, the concomitant discontinuation of insulin delivery upon PN interruption, and the consequently lower risk of hypoglycemic events. The latter is particularly relevant for patients in whom the transient need for exogenous insulin is primarily a result of their nutrition support. However, the need for strict aseptic conditions may render the procedure impractical or not permissible. Furthermore, reservations exist regarding the diminished or highly variable efficacy of PN insulin due to interference from PN ingredients or bag surface material [54,55].

Irrespective to the chosen approach for insulin administration, a limiting factor in obtaining tight glucose control is the risk of inadvertent hypoglycemia [17]. As is the case for hyperglycemia, iatrogenic hypoglycemia is associated with increased cost and adverse medical outcomes [16]. In this context, there is an increasing interest in the use of noninsulin glucose-lowering agents (without hypoglycemia risk) for the treatment of inpatient hyperglycemia. The recent SITA-HOSPITAL randomized controlled study investigated 279 noncritical care patients with type 2 diabetes and showed that oral sitagliptin plus basal insulin led to similar glycemic control than the more labor-intensive basal–bolus insulin regimen [56]. However, challenging patients such as those with high insulin requirements, renal failure, or use of glucocorticoids were excluded from the study. Currently, there are no data on the inpatient use of noninsulin glucose-lowering treatments for hyperglycemia in noncritical care patients on PN and/or EN. Reservations apply to the common side effects of noninsulin treatments (e.g., incretin-based therapies) on the gastrointestinal tract, which is often the primary pathophysiology calling for nutrition support. Further research is needed to explore the potential risks and benefits of noninsulin pharmacotherapy for managing glucose levels in noncritical care settings with EN/PN.

The high prevalence of hyperglycemia amongst noncritical care patients on PN and/or EN support, in conjunction with the increasing workload burden placed on hospital staff, brings an urgent need for innovative approaches to improve the efficacy, efficiency, and safety of healthcare delivery in this context. The advent of novel technologies such as automated closed-loop systems that titrate insulin delivery based on real-time sensor glucose measurements could potentially address this need, whilst reducing staff workload burden. Uncertainties remain with respect to interference with certain medications and inaccurate glucose readouts related to compromised microcirculation. In addition, we concede that there are short-term costs to purchase, install, and train staff in the use of any novel technology. Ongoing studies will further document the potential role of this technology and the obstacles to its integration into clinical practice without disrupting the usual workflow.

**Figure 4:**
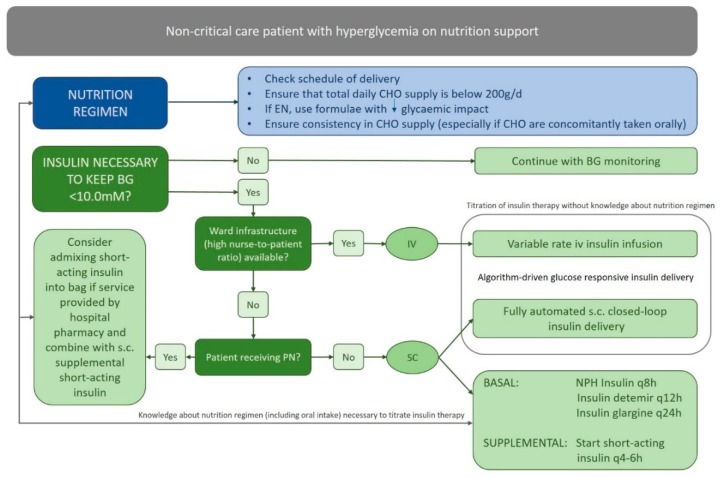
Approach to the management of hyperglycemia in patients receiving enteral or parenteral nutrition. CHO = carbohydrates, EN = enteral nutrition, BG = blood glucose, IV = intravenous, PN = parenteral nutrition, SC = subcutaneous, and q8h = dosing every 8 h.

The present systematic review turned up rather few randomized controlled trials and a limited number of retrospective observational studies addressing hyperglycemia in noncritical care patients on PN and/or EN. The studies were highly heterogeneous in terms of study population, nutrition regimen, study endpoints, and glucose measurement techniques. Patients with pre-existing diabetes already on insulin treatment before their admission to the hospital and those receiving steroids or showing impaired renal function clearly have different optimal insulin delivery profiles compared to patients without pre-existing diabetes and/or with stress-induced hyperglycemia. The particular nutrition regime (i.e., the EN/PN feeding schedule and any additional oral intake) leads to variable carbohydrate exposures with relevant impact on insulin requirements. The few studies that utilized CGM more comprehensively assessed both hyper- and hypoglycemic excursions, whilst studies adopting intermittent (i.e., six-hourly) point-of-care measurements may have missed important events such as postprandial transients. Most studies were conducted in patients on continuous PN and/or EN, which may be less demanding for insulin management compared to the less common bolus or cyclic feeding practices. The scarce evidence and the many factors (e.g., patient comorbidity, staffing level, hospital guidelines, and policies) that determine the ability and capacity to treat hyperglycemia effectively and safely challenge the provision of generalizable treatment recommendations. In Figure 4, we propose a workflow recommendation considering both nutritional and insulin adaptation to manage hyperglycemia in the noncritical care population receiving nutrition support.

The majority of the included studies scored poorly in methodology, with a high or unclear risk of biases according to Cochrane criteria. Undoubtably, it is sometimes difficult or unethical to undertake double-blind RCTs with standardized protocols. Also, some studies did not correct for multiple testing, which may have overestimated outcomes. The included retrospective observational studies were not evaluated for quality given the known risk of outcome overestimation and confounding biases inherent in that design. However, we chose to report these studies in this review to cover the widest possible range of different management possibilities.

Small sample sizes and short study durations of some studies may have led to an underestimation of effect sizes, thus hindering the sensitivity to ascertain the efficacy of potential methods to improve glycemic control or indeed to confirm the impact of superior glucose control on patient outcomes. There is clearly a need for further research in the form of well-designed and adequately powered multicenter trials of sufficient duration aiming to examine effects of glucose management strategies on glucose control, clinical outcome, and also optimization of nutritional status in patients receiving PN and/or EN.

## 5. Conclusions

The management of hyperglycemia in patients receiving PN and/or EN presents unique clinical challenges for both diabetic and nondiabetic hospitalized patients with hyperglycemia. Coherent approaches to this problem are important to avoid potential complications. Obtaining a better match between the carbohydrate dose and the insulin supply is likely to improve glucose control. Granting more attention to the glycemic impact of nutrition regimes in conjunction with deploying novel technologies such as CGM and glucose-responsive automation of insulin delivery through closed-loop systems may address these needs without increasing staff workload. Supplemental or alternate use of noninsulin pharmacological approaches may further open up new lines of research. Well-designed and adequately powered randomized controlled trials are necessary to define the optimal management of hyperglycemia and consequent clinical benefits in patients receiving nutrition support.

## Figures and Tables

**Table 1:** Overview of clinical trials.

Author, Year	Study Design ^1^	Sample Size	Population	Nutrition Therapy	Interventions	Primary Outcome {Study Period}	Main Results	Risk of Bias ^3^
					Insulin adaptation			
Boughton et al., 2019 [31]	RCT, parallel, two-center	43	Non-T1D surgical and medical	PN and/or EN	Fully automated s.c. closed-loop insulin delivery (closed loop, *n* = 21)	% time in target (5.6–10.0 mM) based on CGM values {Up to 15 d or discharge}	% time in target higher in closed-loop vs. control (68% vs. 36%, *p* < 0.0001); hypoglycemia (<3.9 mM) infrequent and similar between closed-loop and control (0.5% for both), *p* = ns)	Low
					Conventional s.c. insulin therapy according to local practice (control, *n* = 22)			
Olveira et al., 2019 [32]	RCT, parallel, multi-center (26 sites)	161	T2D surgical and medical	TPN	100% regular insulin in PN bag (100% in bag, *n* = 80)	Mean glucose based on capillary POC BG values {Up to 15 d or until PN stop}	Mean glucose during TPN 9.2 vs. 9.6 mM 100% in bag vs. 50% in bag (ns); mean glucose 48 h post-TPN higher in 100% in bag vs. 50% in bag (8.9 vs. 7.9 mM, *p* = 0.024); number of patients with hypoglycemia (≤3.9 mM) lower in 100% in bag vs. 50% in bag (9 vs. 22, *p* = 0.016)	Medium
					50% s.c. glargine + 50% regular insulin in PN bag (50% in bag, *n* = 81)			
Li et al., 2018 [38]	RCT, parallel, single-center	102	T2D surgical	PN (cyclic)	Continuous s.c. insulin infusion (CSII, *n* = 50)	mean amplitude of glycemic excursion (MAGE) based on CGM {4 d}	MAGE lower in CSII vs. basbol (3.7 vs. 6.2 mM, *p* < 0.05), no hypoglycemia events (<3.9 mM) occurred	High
					S.c. basal–bolus glargine/aspart (basbol, *n* = 52)			
Hakeam et al., 2017 [33]	RCT, parallel, single-center	67	Non-T1D surgical (non-cardiac)	PN	S.c. glargine (scGlarg, *n* = 35)	Mean glucose based on capillary POC BG values from day 5 on PN and % of patients who achieved target glycaemia (7.8–10.0 mM) {9 d}	Comparable mean BG in scGlar vs. RIbag, % of values in target 52% in scGlar vs. 48% in RIbag (*p* = 0.06); no significant difference in hypoglycemic (<3.9 mM) events	Medium
					Regular insulin added to PN bag (RIbag, *n* = 32)			
Yuan et al., 2015 [12]	RCT, parallel, single-center	212	T2D surgical (gastrectomy for gastric cancer)	EN (continuous)	VRII (short-acting insulin NOS) (VRII, *n* = 106)	PO not specified; mean glucose based on capillary POC BG values, infective and noninfective complications {8–10 d}	Mean BG lower in VRII vs. s.c.Ins. (5.4 vs. 9.5 mM, *p* < 0.001); higher rate of severe hypoglycemia (≤2.2 mM) in VRII vs. s.c.Ins. (8% vs. 1%, *p* = 0.035)	High
					S.c. conventional insulin therapy (s.c.Ins., *n* = 106)			
Kruyt et al., 2010 [39]	Single-arm intervention with historical control	23	Hyperglycemic patients (excluded patients with previous insulin use)medical (stroke unit)	EN	Continuous feeding with computerized VRII group (continuous, *n* = 10)	% of capillary POC BG values in target range (4.4–6.1 mM){5 d}	Higher % values in target and mean glucose in continuous vs. inter group (55% vs. 19%, *p* < 0.005 and 5.8 vs. 7.6 mM, *p* < 0.005)	Medium
					Bolus feeding with regular i.v. insulin adaptation intermediate group (inter, *n* = 13)			
Korytkowski et al., 2009 [40]	RCT, parallel, single-center	50	Diabetes NOS surgical and medical	EN	S.c. SSI (regular insulin) every 4–6 h (SSRI, *n* = 25); (NPH initialized if persistent BG > 10.0 mM)	PO not specified; mean glucose based on capillary POC BG values {8 d}	mean BG similar in SSRI and basalPLUS (8.9 vs. 9.2 mM, *p* = ns); NPH initialized in 55% of those on SSRI	High
					S.c. SSRI plus s.c. glargine (basalPLUS, *n* = 25)			
					Nutrition adaptation			
Tiyapanjanit et al., 2014 [35]	Non-randomized cross-over (no washout)	10	T2D (BG < 10 mM wo antidiabetic medication) medical	EN (continuous)	In-house prepared EN formula with 50% CHO thereof 67% fructose, (inhouse, *n* = 10)	PO not specified; mean glucose based on capillary POC BG values; glycemic variability based on CGM {3 d, each formula for 36 h)	Mean glucose lower in inhouse vs. standard group (6.8 vs. 8.0 mM, *p* = 0.022); glucose variability comparable	High
					Standard diabetic EN formula with 53% CHO thereof 15% fructose and 57% maltodextrin (standard, *n* = 10)			
Lidder et al., 2009 [34]	RCT, parallel, single-center	30	Prediabetes (fasting BG < 7 mM) surgical (esophagectomy for esophageal cancer)	PN with or wo EN	100% of energy covered by PN (PN, *n* = 14)	Mean glucose values based on CGM values {surgery until postoperative day 5}	Mean glucose comparable over entire study period, lower from day 3 post-surgery to day 5 post-surgery in PN + EN vs. PN (*p* = 0.002)	Medium
					70% covered by PN + 30% covered by EN (PN + EN, *n* = 16)			
Léon-Sanz et al., 2005 [36]	RCT, parallel, multi-center (4 sites)	104	T2D surgical and medical	EN (continuous)	Low-CHO high-MUFA nutrition formula (lowCHO, *n* = 51)	Mean glucose based on capillary POC BG values and mean daily insulin dose {2 weeks}	BG increase from baseline lower in lowCHO than highCHO after 7 d on EN (10% vs. 21%, *p* = 0.006); mean BG identical (12.7 vs. 12.7 mM^2^)	Medium
					Energy-matched high-CHO nutrition formula (highCHO, *n* = 53)			
Valero et al., 2001 [41]	RCT, parallel, double-blind, single-center	138	T1D (21%) and T2D (79%) surgical and medical	TPN (continuous)	Standard TPN containing glucose (PN_G, *n* = 71)	PO not specified; number of patients with target glycaemia (capillary POC BG values 8.3–11.1 mM) at end of TPN {TPN duration 5–46 d)	BG < 11.1 mM at end of TPN reached in 75% vs. 85% in PN_G and PN_GFX respectively ^2^	High
					Energy-matched TPN containing glucose:fructose:xylitol 2:1:1 (PN_GFX, *n* = 67)			

RCT = randomized controlled trial, T1D = type 1 diabetes, EN = enteral nutrition, PN = parenteral nutrition, s.c. = subcutaneous, CGM = continuous glucose monitoring, T2D = type 2 diabetes, TPN = total parenteral nutrition, POC = point of care, BG = blood glucose, (ns) = not significant, CSII = continuous subcutaneous insulin infusion, VRII = variable rate intravenous insulin, excl = exclusion, NOS = not otherwise specified, PO = primary outcome, SSI = sliding scale insulin, SSRI = sliding-scale regular insulin, NPH = neutral protamine Hagedorn insulin, wo = without, i.v. = intravenous, CHO = carbohydrates, h = hours, d = day(s) and MUFA = monounsaturated fatty acids. ^1^ Open-label if not stated otherwise. ^2^ No *p*-value available. ^3^ Overall quality assessment; specific domains can be found in Supplementary Figure S1.

**Table 2:** Overview of observational, retrospective studies.

Author, Year	Study Design	Sample Size	Population	Nutrition Therapy	Interventions	Primary Outcome {Study Period}	Main Results
					Insulin adaptation		
Truong et al., 2019 [42]	Retrospective observational, single-center	102	Hyperglycemic patients (≥ 2 BG values > 10.0 mM, including T1D and T2D) surgical and medical	PN (continuous or cyclic)	Regular insulin added to PN bag (100% bag, *n* = 78)	% of patients with ≥5/6 capillary POC BG values per day <10.0 mM for ≥2 consecutive days {during PN or until target reached}	Higher % of patients with target achieved in 100%bag vs. scGlarg (72% vs. 49%, *p* = 0.017); ≥2 hypoglycemic events (<3.9 mM) in 9% of 100%bag and 17% of scGlarg group (*p* = ns); lower need for corrective insulin less in 100%bag vs. scGlarg (28 vs. 57% of patients, *p* = 0.003)
					S.c. insulin glargine (scGlarg, *n* = 35)		
Hijaze et al., 2017 [43]	Retrospective observational, single-center	53	Non-T1D medical	EN (continuous)	S.c. NPH insulin 3×/day (NPH, *n* = 26) (Rescue bolus with 6 units rapid insulin analoga if BG > 16.7 mM)	PO not specified; mean glucose based on capillary POC BG values and % of values in the target range (7.8–10 mM) {until discharge or stop EN}	mean BG comparable in NPH vs. basbol (10.6 vs. 11.1 mM, *p* = ns), 24% and 22% of values in range (*p* = ns)
					S.c. basal–bolus insulin analoga therapy (basbol, *n* = 27)		
Neff et al., 2014 [44]	Retrospective observational, single-center	53	Hyperglycemic patients (BG > 10 mM including T1D and T2D) surgical and medical	PN	Protocol-driven VRII (VRII, *n* = 32)	PO not specified; mean glucose based on POC BG values and % of values in target range (4.0–10.0 mM) {until stop PN}	Mean glucose lower and % values in target range higher in VRII vs. basbol (9.6 vs. 11.2 mM, *p* = 0.009 and 62% vs. 43%, *p* = 0.008)
					S.c. basal–bolus insulin (basbol, *n* = 21)		
Hsia et al., 2011 [45]	Retrospective observational, single-center	22	Diabetes NOS surgical and medical	EN (continuous)	S.c. basal–bolus with glargine/lispro (basbol, *n* = 8)	PO not specified; mean glucose based on POC BG values, % of values in target range (7.8–10.0 mM) {72 h}	mean glucose comparable, % of values in target range higher in mixed3 vs. mixed2 and basbol (69% vs. 22% vs. 24%, *p* < 0.01); patients with hypoglycemic events (<3.9 mM) in basbol vs. mixed2 and mixed3 (5 vs. 2 vs. 1)
					S.c. 70/30 premixed insulin 2×/day (mixed2, *n* = 8)		
					S.c. 70/30 pre-mixed insulin 3×/day (mixed3, *n* = 6)		

BG = blood glucose, vs. = versus, T1D = type 1 diabetes, T2D = type 2 diabetes, PN = parenteral nutrition, s.c. = subcutaneous, POC = point of care, EN = enteral nutrition, NPH = Neutral Protamine Hagedorn, ns = not significant, VRII = variable rate intravenous insulin, PO = primary outcome, and NOS = not otherwise specified.

## Supplementary Materials

The following are available online at https://www.mdpi.com/2077-0383/8/7/935/s1; Figure S1: Quality assessment of clinical trials according to modified Cochrane Risk of Bias tool [30]. Table S1: PubMed Search Strategy 12.04.2019, Table S2: Embase Search Strategy 10.04.2019, Table S3: Cochrane Central Register of Controlled Trials Search Strategy 10.04.2019, Table S4: ClinicalTrials.gov Search Strategy 10.04.2019, Table S5: PRISMA 2009 checklist [28], Table S6: MOOSE checklist [29].
