# Supplementary material for: Management of Glucose Control in Noncritically Ill, Hospitalized Patients Receiving Parenteral and/or Enteral Nutrition: A Systematic Review
Source: J Clin Med. 2019 Jun 28;8(7):935. doi: 10.3390/jcm8070935 (PMC6678336; doi:10.3390/jcm8070935)
Supplement: Supplementary file 1 [file jcm-08-00935-s001.pdf]

**Table S1.** PubMed Search Strategy 12.04.2019.

| Search | Query | Results |
|---|---|---|
| #10 | #9 Filters: Publication date from 1999/04/10 to 2019/04/10 | 443 |
| #9 | #7 OR #8 | 650 |
| #8 | #5 AND #6 | 360 |
| #7 | #1 AND #2 AND #3 AND #4 | 440 |
| #6 | (((“Enteral Nutrition)” [MeSH:NoExp]) OR “Parenteral Nutrition”[MeSH:NoExp]) OR “Parenteral Nutrition, Total”[MeSH:NoExp] | 37188 |
| #5 | (“Hyperglycemia” [MeSH]) OR (“Diabetes Mellitus” [MeSH]) | 415262 |
| #4 | (Blood glucose* OR Glycaemi* OR Glycemi* OR glucose control* OR glucose monitoring* OR glucose management* OR closed loop* OR closed-loop) | 224471 |
| #3 | (Inpatient* OR “patients” [tiab] OR Hospitalization OR Hospitalisation OR hospitalized OR hospitalized) | 5478370 |
| #2 | (hyperglycemia OR hyperglycaemia OR diabetic OR hyperglycemic) | 292983 |
| #1 | (nutrition support* OR medical nutrition therap* OR artificial feeding* OR tube feeding* OR gastric feeding* OR enteral nutrition* OR parenteral nutrition* OR parenteral feeding* OR intravenous feeding* OR parenteral hyperalimentation*) | 51508 |

**Table S2.** Embase Search Strategy 10.04.2019.

| Search | Query | Results |
|---|---|---|
| #6 | #5 AND [embase]/lim NOT ([embase]/lim AND [medline]/lim) | 99 |
| #5 | #1 AND #2 AND #3 AND #4 | 238 |
| #4 | 'glucose blood level'/exp OR 'blood glucose':ti,ab,kw OR 'glycaemi*':ti,ab,kw OR 'glycemi*':ti,ab,kw OR 'glucose control':ti,ab,kw OR 'glucose management':ti,ab,kw OR 'glucose monitoring':ti,ab,kw OR 'closed loop':ti,ab,kw OR 'closed-loop':ti,ab,kw | 326057 |
| #3 | 'hospital patient'/exp OR 'hospital patient':ti,ab,kw OR 'inpatient':ti,ab,kw OR 'hospitalisation':ti,ab,kw OR 'hospitalization':ti,ab,kw OR 'hospitalised':ti,ab,kw OR 'hospitalized':ti,ab,kw | 481450 |
| #2 | 'enteric feeding'/de OR 'enteric feeding':ti,ab,kw OR 'nose feeding'/de OR 'nose feeding':ti,ab,kw OR 'parenteral nutrition'/exp OR 'parenteral nutrition':ti,ab,kw OR 'nutrition supplement'/de OR 'nutrition support':ti,ab,kw OR 'medical nutrition therap*':ti,ab,kw OR 'artificial feeding':ti,ab,kw OR 'tube feeding':ti,ab,kw OR 'enteral nutrition':ti,ab,kw OR 'gastric feeding':ti,ab,kw OR 'parenteral nutrition'/de OR 'total parenteral nutrition'/de OR 'parenteral feeding':ti,ab,kw OR 'intravenous feeding':ti,ab,kw OR 'intragastric feeding':ti,ab,kw OR 'parenteral hyperalimentation':ti,ab,kw | 84264 |
| #1 | 'hyperglycemia'/de OR 'hyperglycemia':ti,ab,kw OR 'hyperglycaemia':ti,ab,kw OR 'hyperglucemia':ti,ab,kw OR 'diabetes mellitus'/de OR 'diabetes mellitus':ti,ab,kw OR diabetic:ti,ab,kw | 863713 |

**Table S3.** Cochrane Central Register of Controlled Trials Search Strategy 10.04.2019.

| Search | Query | Results |
|---|---|---|
| #15 | Not listed in PubMed or Embase | 62 |
| #14 | Filtered for years 1999-2019 and Trials | 550 |
| #13 | #9 OR #12 | 774 |
| #12 | #10 AND #11 | 58 |
| #11 | #7 OR #8 | 27862 |
| #10 | #5 OR #6 | 2381 |
| #9 | #1 AND #2 AND #3 AND #4 | 756 |
| #8 | MeSH descriptor: [Diabetes Mellitus] explode all trees | 26838 |
| #7 | MeSH descriptor: [Hyperglycemia] explode all trees | 2602 |
| #6 | MeSH descriptor: [Hyperglycemia] explode all trees | 760 |
| #5 | MeSH descriptor: [Enteral Nutrition] explode all trees | 1713 |
| #4 | Blood glucose* OR Glycaemi* OR Glycemi* OR glucose control* OR glucose monitoring* OR glucose management* OR closed loop* OR closed-loop | 62566 |
| #3 | (Inpatient* OR patients OR Hospitalization OR Hospitalisation OR hospitalized OR hospitalised) | 824802 |
| #2 | (hyperglycemia OR hyperglycaemia OR diabetic OR hyperglycemic) | 38420 |
| #1 | (nutrition support* OR medical nutrition therap* OR artificial feeding* OR tube feeding* OR gastric feeding* OR enteral nutrition* OR parenteral nutrition* OR parenteral feeding* OR intravenous feeding* OR parenteral hyperalimentation*) | 30100 |

**Table S4.** ClinicalTrials.gov Search Strategy 10.04.2019.

| Search | Query | Results |
|---|---|---|
| #1 | Glucose AND (parenteral OR enteral Nutrition) | 141 |

| | Random sequence generation | Allocation concealment | Selective reporting | Other sources of bias | Performance bias | Detection bias | Attrition bias |
|---|---|---|---|---|---|---|---|
| Boughton et al., 2019 | + | + | ? | + | − | − | + |
| Olveira et al., 2019 | + | + | ? | + | − | − | + |
| Li et al. 2018 | ? | ? | ? | + | − | − | ? |
| Hakeam et al., 2017 | + | + | ? | + | − | − | + |
| Yuan et al., 2015 | ? | ? | ? | − | − | − | − |
| Korytkowski et al., 2009 | ? | ? | − | − | − | − | ? |
| Lidder et al., 2009 | + | + | ? | ? | − | + | + |
| Léon-Sanz et al., 2005 | + | + | ? | + | − | − | − |
| Valero et al., 2001 | ? | ? | ? | − | + | − | ? |
| Tiyapanjanit et al., 2014 | N/A | N/A | ? | − | N/A | N/A | ? |
| Kruyt et al., 2001 | N/A | N/A | ? | + | N/A | N/A | + |

**Key**

? Unclear Risk of bias

+ Low Risk of bias

− High Risk of bias

N/A Not applicable

Other sources of bias defined as definition of primary outcome or correction for multiple testing

**Figure S1.** Quality assessment of clinical trials according to modified Cochrane Risk of Bias tool [30].

**Table S5.** PRISMA 2009 checklist [28].

| Section/Topic | # | Checklist Item | Reported on Page # |
|---|---|---|---|
| | | **TITLE** | |
| Title | 1 | Identify the report as a systematic review, meta-analysis, or both. | 1 |
| | | **ABSTRACT** | |
| Structured summary | 2 | Provide a structured summary including, as applicable: background; objectives; data sources; study eligibility criteria, participants, and interventions; study appraisal and synthesis methods; results; limitations; conclusions and implications of key findings; systematic review registration number. | 1 |
| | | **INTRODUCTION** | |
| Rationale | 3 | Describe the rationale for the review in the context of what is already known. | 1–3 |
| Objectives | 4 | Provide an explicit statement of questions being addressed with reference to participants, interventions, comparisons, outcomes, and study design (PICOS). | 3 |
| | | **METHODS** | |
| Protocol and registration | 5 | Indicate if a review protocol exists, if and where it can be accessed (e.g., Web address), and, if available, provide registration information including registration number. | N/A |
| Eligibility criteria | 6 | Specify study characteristics (e.g., PICOS, length of follow-up) and report characteristics (e.g., years considered, language, publication status) used as criteria for eligibility, giving rationale. | 3 |
| Information sources | 7 | Describe all information sources (e.g., databases with dates of coverage, contact with study authors to identify additional studies) in the search and date last searched. | 3 |
| Search | 8 | Present full electronic search strategy for at least one database, including any limits used, such that it could be repeated. | Supplementary Tables S1–S4 |
| Study selection | 9 | State the process for selecting studies (i.e., screening, eligibility, included in systematic review, and, if applicable, included in the meta-analysis). | 3–4 |
| Data collection process | 10 | Describe method of data extraction from reports (e.g., piloted forms, independently, in duplicate) and any processes for obtaining and confirming data from investigators. | 4 |
| Data items | 11 | List and define all variables for which data were sought (e.g., PICOS, funding sources) and any assumptions and simplifications made. | 4 |
| Risk of bias in individual studies | 12 | Describe methods used for assessing risk of bias of individual studies (including specification of whether this was done at the study or outcome level), and how this information is to be used in any data synthesis. | 4 |
| Section/topic | # | Checklist item | Reported on page # |
| Summary measures | 13 | State the principal summary measures (e.g., risk ratio, difference in means). | N/A |
| Synthesis of results | 14 | Describe the methods of handling data and combining results of studies, if done, including measures of consistency (e.g., I2) for each meta-analysis. | N/A |
| Risk of bias across studies | 15 | Specify any assessment of risk of bias that may affect the cumulative evidence (e.g., publication bias, selective reporting within studies). | 5 |
| Additional analyses | 16 | Describe methods of additional analyses (e.g., sensitivity or subgroup analyses, meta-regression), if done, indicating which were pre-specified. | N/A |
| | | **RESULTS** | |
| Study selection | 17 | Give numbers of studies screened, assessed for eligibility, and included in the review, with reasons for exclusions at each stage, ideally with a flow diagram. | 4–5 |

| | | | |
|---|---|---|---|
| Study characteristics | 18 | For each study, present characteristics for which data were extracted (e.g., study size, PICOS, follow-up period) and provide the citations. | 5–8 |
| Risk of bias within studies | 19 | Present data on risk of bias of each study and, if available, any outcome level assessment (see item 12). | Supplementary Figure S1 |
| Results of individual studies | 20 | For all outcomes considered (benefits or harms), present, for each study: (a) simple summary data for each intervention group (b) effect estimates and confidence intervals, ideally with a forest plot. | Tables 1 and 2 |
| Synthesis of results | 21 | Present results of each meta-analysis done, including confidence intervals and measures of consistency. | N/A |
| Risk of bias across studies | 22 | Present results of any assessment of risk of bias across studies (see Item 15). | Supplementary Figure S1 |
| Additional analysis | 23 | Give results of additional analyses, if done (e.g., sensitivity or subgroup analyses, meta-regression). | N/A |
| | | **DISCUSSION** | |
| Summary of evidence | 24 | Summarize the main findings including the strength of evidence for each main outcome; consider their relevance to key groups (e.g., healthcare providers, users, and policy makers). | 12–13 |
| Limitations | 25 | Discuss limitations at study and outcome level (e.g., risk of bias), and at review-level (e.g., incomplete retrieval of identified research, reporting bias). | 12–13 |
| Conclusions | 26 | Provide a general interpretation of the results in the context of other evidence, and implications for future research. | 13 |
| | | **FUNDING** | |
| Funding | 27 | Describe sources of funding for the systematic review and other support (e.g., supply of data); role of funders for the systematic review. | 14 |

**Table S6.** MOOSE checklist [29].

| | Criteria | Brief Description of How the Criteria were Handled in the Systematic Review |
|---|---|---|
| | | **Reporting of Background should Include** |
| √ | Problem definition | Hyperglycaemia is frequently encountered in patients receiving artificial nutrition, and optimal glucose management is challenging. |
| √ | Hypothesis statement | None. |
| √ | Description of study outcomes | Outcomes concerning glucose management such as mean glucose values. |
| √ | Type of exposure or intervention used | Pharmacological or nutritional strategies used to manage hyperglycaemia. |
| √ | Type of study designs used | We included clinical trials and observational studies comparing two different interventions. |
| √ | Study population | Adult individuals from the general population with hyperglycaemia, with or without diabetes. |
| | | **Reporting of search strategy should include** |
| √ | Qualifications of searchers | The credentials of the investigators are indicated in the authors list. |
| √ | Search strategy, including time period included in the synthesis and keywords | Search strategy and time periods are described in page 3 of the manuscript and in Supplementary Tables S1–S4. |
| √ | Databases and registries searched | Embase.com, PubMed, Cochrane Central Register of Controlled Clinical Trials and ClinicalTrials.gov |
| √ | Search software used, name and version, including special features | We did not employ any search software. EndNote was used to merge retrieved citations. |
| √ | Use of hand searching | We did not hand-search bibliographies of retrieved papers. |
| √ | List of citations located and those excluded, including justifications | Details of the literature search and selection process are outlined in the flow chart on page 4. Citations for the included studies are enclosed in the Table 1 and 2. The citation list for excluded studies is available upon request. |
| √ | Method of addressing articles published in languages other than English | We included records published or registered exclusively in English. |
| √ | Method of handling abstracts and unpublished studies | Abstracts were excluded, unpublished studies were screened, one of them fit the inclusion criteria. Since there are no results available, this study was not included in Table 1 or Table 2. |
| √ | Description of any contact with authors | None. |
| | | **Reporting of methods should include** |
| √ | Description of relevance or appropriateness of studies assembled for assessing the hypothesis to be tested | Detailed inclusion and exclusion criteria are described in the Methods section. |
| √ | Rationale for the selection and coding of data | Data extracted from each of the studies were relevant to the population characteristics, study design, glucose management strategy, sample size and primary outcome as well as main results deemed relevant. |
| √ | Assessment of confounding | This review included clinical trials. Since retrospective publications have an inherently high risk of bias in confounding, we refrained them from a quality evaluation. |
| √ | Assessment of study quality, including blinding of quality assessors; stratification or regression on possible predictors of study results | Clinical trials quality was assessed using the Cochrane risk of bias tool. |
| √ | Assessment of heterogeneity | We did not perform any test of heterogeneity. |
| √ | Description of statistical methods in sufficient detail to be replicated | We did not perform statistical analyses. |
| √ | Provision of appropriate tables and graphics | We included 2 main tables. |
| | | **Reporting of results should include** |
| √ | Graph summarizing individual study estimates and overall estimate | N/A |
| √ | Table giving descriptive information for each study included | Table 1 and Table 2. |
| √ | Results of sensitivity testing | N/A |
| √ | Indication of statistical uncertainty of findings | N/A. |

| | | |
|---|---|---|
| **Reporting of discussion should include** | | |
| √ | Quantitative assessment of bias | We did not perform a quantitative assessment of bias. |
| √ | Justification for exclusion | We excluded studies that did not compare the same exposure or outcome assessment in both groups. |
| √ | Assessment of quality of included studies | Study quality was assessed using the Cochrane risk of bias tool. Retrospective studies were refrained from quality evaluation. |
| **Reporting of conclusions should include** | | |
| √ | Consideration of alternative explanations for observed results | We discussed that multiple testing might be an alternative explanation for the observed outcomes, as not all studies corrected for this or defined primary outcome. |
| √ | Generalization of the conclusions | The generalizability of our findings has been limited by the small sample sizes of the included trials. Also, the findings are not generalizable to children as the included trials only comprised the adult population. |
| √ | Guidelines for future research | Future studies are required to determine the best management option for glucose control in larger populations. |
| √ | Disclosure of funding source | No separate funding was necessary for the undertaking of this systematic review. |
